# Alternative approaches to analyzing ventilator-free days, mortality and duration of ventilation in critical care research

**DOI:** 10.62675/2965-2774.20240246-en

**Published:** 2024-05-07

**Authors:** Ary Serpa, Michael Bailey, Yahya Shehabi, Carol L Hodgson, Rinaldo Bellomo

**Affiliations:** 1 Monash University School of Public Health and Preventive Medicine Australian and New Zealand Intensive Care Research Centre Melbourne Victoria Australia Australian and New Zealand Intensive Care Research Centre, School of Public Health and Preventive Medicine, Monash University - Melbourne, Victoria, Australia.; 2 Hospital Israelita Albert Einstein Department of Critical Care Medicine São Paulo SP Brazil Department of Critical Care Medicine, Hospital Israelita Albert Einstein - São Paulo (SP), Brazil.; 3 Monash University Monash Health School of Clinical Sciences Melbourne Victoria Australia Monash Health School of Clinical Sciences, Monash University - Melbourne, Victoria, Australia.; 4 Austin Hospital Department of Intensive Care Heidelberg VIC Australia Department of Intensive Care, Austin Hospital - Heidelberg VIC, Australia.

**Keywords:** Critical care outcomes, Methods, Statistics, Respiration, artificial, Critical care

## Abstract

**Objective:**

To discuss the strengths and limitations of ventilator-free days and to provide a comprehensive discussion of the different analytic methods for analyzing and interpreting this outcome.

**Methods:**

Using simulations, the power of different analytical methods was assessed, namely: quantile (median) regression, cumulative logistic regression, generalized pairwise comparison, conditional approach and truncated approach. Overall, 3,000 simulations of a two-arm trial with n = 300 per arm were computed using a two-sided alternative hypothesis and a type I error rate of α = 0.05.

**Results:**

When considering power, median regression did not perform well in studies where the treatment effect was mainly driven by mortality. Median regression performed better in situations with a weak effect on mortality but a strong effect on duration, duration only, and moderate mortality and duration. Cumulative logistic regression was found to produce similar power to the Wilcoxon rank-sum test across all scenarios, being the best strategy for the scenarios of moderate mortality and duration, weak mortality and strong duration, and duration only.

**Conclusion:**

In this study, we describe the relative power of new methods for analyzing ventilator-free days in critical care research. Our data provide validation and guidance for the use of the cumulative logistic model, median regression, generalized pairwise comparisons, and the conditional and truncated approach in specific scenarios.

## INTRODUCTION

The number of ventilator-free days (VFDs) is one of several organ failure-free outcomes commonly used in critical care research, especially in studies focused on respiratory system-directed interventions.^(1)^ Ventilator-free days represents a composite outcome that combines both mortality and duration of ventilation into a single variable, thus attenuating the effect of the competing risk of mortality. A key rationale behind VFDs is to have a continuous outcome that provides greater statistical power to detect a treatment effect than binary outcomes alone. In a recent paper, Yehya et al. provided a thorough framework for determining when and how to use VFDs, along with a comprehensive discussion of the different methods for analysis and interpretation and the relative statistical power of each test.^(1)^ In this regard, recent studies have also explored additional methods of analysis, namely, quantile (median) regression,^(2,3)^ cumulative logistic regression,^(4,5)^ generalized pairwise comparisons, including the win ratio method,^(6)^ and conditional and truncated approaches.

In this study, we seek to introduce the concept of the perception distortion effect, further discuss additional aspects of the use of VFDs in critical care research, and build on previous work by considering the relative power of additional approaches. In addition, power simulations based on a previous study and alternative methods for analysis were tested and described.

## PERCEPTION DISTORTION EFFECT

The perception distortion effect relates to the way clinicians perceive and react differently to the findings of a given intervention according to the way it is presented. For example, we consider an intervention that has not affected mortality (identical in both groups) but has decreased the duration of ventilation by one day in a population of patients with an average duration of ventilation of two days. Thus, the mean duration in the control group was 3 days, and in the intervention group, it was 2 days. Most clinicians would react to this finding as a substantial improvement with clinical and practice implications. Currently, these patients are followed up for 28 days, and the outcome of VFD is expressed as the median, which would not be influenced in any meaningful way by even 20% mortality. The findings would be 25 *versus* 26 VFDs.

This would be seen as trivial and would likely not trigger nearly the same response. In the minds of clinicians, the former would be seen as a 33% improvement; the latter would be seen as a 3.8% improvement. This distortion is even more dramatic if the follow-up is extended to 90 or 180 days. In this way, VFDs may distort the perception and reaction to a major effect on the duration of ventilation, resulting in dismissal and neglect of therapies that have achieved such effects. This suggests that combining VFDs as an outcome with the additional outcome of duration of ventilation in survivors may be advantageous in the absence of a numerical increase in mortality among patients receiving the intervention being assessed.

In medicine, cognitive biases such as perception distortion result in diagnostic errors and delays in the acceptance of new scientific findings. For example, despite good evidence suggesting the impact of serum human leukocyte antigen (HLA) antibodies on transplant outcomes, routine inclusion of HLA antibody testing as part of posttransplant monitoring has not been a consensus recommendation for more than 30 years.^(7)^ In addition, responses to the detection of HLA antibodies in the serum continue to vary, and a consensus recommendation for routine treatment has not been reached for more than 40 years. This delay in the acceptance of the role of HLA antibodies in transplant rejection is an example of a cognitive bias such as confirmation bias or perception distortion of research findings.^(7)^

## ALTERNATIVE APPROACHES TO ANALYSIS

### Quantile (median) regression

Since its inception in 1978,^(8)^ quantile or median regression has become an important tool in medical research for the analysis of nonparametric data and has offered a similar advantage of enabling covariate adjustment and treatment effect estimates with confidence intervals. However, due to the composite and ranking nature of VFDs, core differences in outcomes can occur even if the median values are identical.^(1)^ In addition, the mortality component is critically important but has little effect on the median. Thus, the power of median regression is likely highly influenced by which component drives the effect of VFDs: the duration of ventilation or mortality.

Quantile regression has many advantages, but its major disadvantage is that its parameters are more difficult to estimate than those of more traditional methods (e.g., Gaussian or generalized regression). Inferences from such quantile regression can be complicated because the estimators for coefficients are not available in closed form.^(9)^ The most common way to address this problem is by using a linear optimization algorithm with confidence intervals based on piecewise linear approximations.^(8)^ Another possible way is to use boosting algorithms. However, the implementation of p values and confidence intervals of the estimated regression parameters is not straightforward.^(10)^ Finally, a more recently developed algorithm was based on asymmetric Laplace likelihood.^(11)^ Thus, estimation could be highly dependent on the method chosen. This method of analysis was recently used in two randomized clinical trials in the critical care field.^(2,3)^

### Cumulative logistic regression

Cumulative logistic regression considers the ranking and ordinal structure of VFDs.^(4,5)^ In this model, the cumulative log odds are modeled such that a parameter greater than 0 reflects an increase in the cumulative odds for the VFD outcome, which implies benefit. A potential advantage of this model is that, with multinomial sampling of independent subjects, the score test statistic from the model is similar to the Wilcoxon rank-sum test statistic,^(12)^ one of the most powerful tests for analyzing VFDs in a variety of scenarios.^(1)^ However, with the cumulative logistic model, it is possible to further adjust for confounders and to extract an effect estimate with a confidence interval. The potential disadvantage is that the model assumes proportional effects across the ordinal VFD scale. This is called the "proportional odds assumption" or the "parallel regression assumption". This method of analysis was recently used in two randomized clinical trials in the critical care field.^(4,5)^

### Generalized pairwise comparison

The number of VFDs is a composite outcome considering the number of deaths and duration of ventilation in the calculation. In clinical practice, the importance of death is much greater than that of the duration of ventilation. When comparing two patients undergoing a new treatment or strategy, it is reasonable to prioritize the effect on death ahead of the effect on the duration of ventilation. Thus, based on this rationale, first, it must be determined whether one died before assessing the duration of ventilation. If that is not known, then one would determine which patient experienced a longer duration of ventilation. If both patients survived and had the same duration of ventilation, they would be considered as tied. This type of analysis is possible in several ways, including the comparison of matched pairs (using a win ratio approach)^(13)^ or unmatched pairs (using the method described by Finkelstein and Schoenfeld).^(14)^ This method of analysis was recently used in one randomized clinical trial in critical care.^(6)^

### Conditional approach

Based on the rationale described above, which prioritizes death over the duration of ventilation, another potential strategy is to use a conditional approach. Such an approach follows a predefined fixed-testing sequence based on clinical information.^(15)^ With this strategy, if the intervention studied simply results in a numerically greater percentage of deaths than in controls, no further assessment is made, and the study is judged as *neutral* or *negative* depending on the magnitude of the effect on mortality. However, if the intervention results in a lower mortality rate, the duration of ventilation in survivors will then be compared between the studied groups by means of traditional tests. This is based on the idea that an intervention leading to a numerical increase in mortality, even if not statistically significant, is of less importance and probably would not be implemented in clinical care even if it resulted in a shorter duration of ventilation. In the present study, we use a hierarchical *t* test and a hierarchical Wilcoxon rank-sum test as conditional approaches.

### Truncated approach

Recently, a novel high-power test for continuous outcomes truncated by death was reported.^(16)^ This approach incorporates the concept that this type of outcome is, in fact, a two-dimensional outcome and that the constructed combined outcome follows a continuous-singular mixture distribution. Based on this assumption, the authors suggest that this unusual distribution is why one cannot resort to nonparametric Wilcoxon rank-sum tests. This is because the singular component of the distribution of the combined outcome will be reduced to simple ties. In this regard, the handling of ties in standard statistical software varies and is opaque. However, the handling of ties is not the main reason why Wilcoxon suffers power loss. The main reason is that the null hypothesis in these Wilcoxon-type tests (stochastic domination) does not handle the empirical fact that treatments might influence mortality and duration of ventilation differently.

The authors propose to model the binary component (i.e., survival) and the continuous part (i.e., actual ventilator-free days) separately but to conduct a single test for no treatment effect on either. This approach provides a single p value for the hypothesis of no treatment effect on the extended ventilator-free days where death is given the lowest possible score. To accommodate potential nonnormality of the recorded ventilator-free days, we describe both the parametric and the semiparametric tests.

## METHODS

To maintain consistency and facilitate comparison, we adopted the same strategy implemented previously.^(1)^ Overall, 3,000 simulations of a two-arm trial with n = 300 per arm were computed using a two-sided alternative hypothesis and a type I error rate of α = 0.05. Mortality was simulated according to a Bernoulli distribution, and the duration of ventilation among survivors was simulated according to an exponential distribution. All deaths were assigned 0 VFDs. Patients with a duration of ventilation longer than 28 days were assigned 0 VFDs, while for the remaining patients, the duration of ventilation was calculated as 28. As previously described,^(1)^ we considered a range of scenarios with varying treatment effects for both mortality and ventilator duration. For comparison and validity, we replicated the power calculations previously performed,^(1)^ including the Fine-Gray competing risk model, Gray test, Wilcoxon rank-sum test, Student's *t* test and Fisher's exact test. For the median regression, we tested three different algorithms: asymmetric Laplace distribution, simplex, and interior point. For the cumulative logistic regression, the VFDs were rounded to one decimal to improve computational efficiency. The win ratio approach was calculated with death prioritized over VFDs in survivors and using the large sample distribution of certain multivariate multisample U-statistics.

All simulations were performed in R v.4.0.2, and the following packages were used in addition to the base program: *lqmm*,^(11)^ *quantreg*,^(17)^ *cmprsk*,^(18)^ *ordinal*,^(19)^ and *WinRatio*.^(20)^ To illustrate the studied methods for analyzing VFDs, two clinical trials were performed: SPICE III^(3)^ and TEAM.^(21)^

## RESULTS

When considering power, median regression did not perform well in studies where the treatment effect was mainly driven by mortality (Table 1). Median regression performed better in situations with a weak effect on mortality but a strong effect on duration, duration only, and moderate mortality and duration. However, the median regression did not perform better than the Wilcoxon rank-sum test in any of these scenarios. The underlying algorithm also plays an important role in determining the power of median regression, with the ‘interior point' algorithm having the greatest power, while the asymmetric Laplace algorithm was the least powerful. The only scenario in which median regression presented the highest power with the asymmetric Laplace algorithm was the conflicting scenario.

**Table 1:** Power calculations for different statistical tests with ventilator-free days on Day 28 as the outcome

Effect		Mortality*(%)	Duration of ventilation in survivors*	Fine-Gray model(%)	Gray's test(%)	Wilcoxon rank-sum test†(%)	Student's t test (%)	Fisher's exact test‡(%)	Median regression§(%)			Cumulative logistic model
									Asymmetric Laplace	Simplex	Interior point	
Mortality only												
	Treatment	15	7.0	77	78	56	72	84	4	12	20	56
	Control	25	7.0									
Strong mortality and weak duration												
	Treatment	15	6.0	94	95	89	94	84	36	52	52	89
	Control	25	7.0									
Moderate mortality and duration												
	Treatment	15	5.0	78	80	85	81	32	24	72	80	85
	Control	20	6.5									
Weak mortality and strong duration												
	Treatment	15	5.0	83	84	97	90	5	44	88	92	97
	Control	16	8.0									
Duration only												
	Treatment	15	5.0	77	78	96	86	4	36	88	92	96
	Control	15	8.0									
Conflicting												
	Treatment	15	6.5	5	5	14	5	32	64	28	24	14
	Control	20	5.0									

The test with the highest power in each scenario is highlighted in bold.

All results based on 3,000 simulated trials with 300 subjects in each of two treatment groups, a two-sided alternative hypothesis, and a type I error rate of α = 0.05.

* Mortality simulated according to a Bernoulli distribution and duration of ventilation in survivors according to an exponential distribution;

† normal approximation with continuity correction was used for the Wilcoxon rank-sum test;

‡ outcome is mortality, duration of ventilation is ignored;

§ all *p* values extracted via bootstrap with 1,000 samples.

When considering power, the cumulative logistic regression was found to produce similar power to the Wilcoxon rank-sum test across all scenarios, being the best strategy for the scenarios of moderate mortality and duration, weak mortality and strong duration, and duration only.

When considering the generalized pairwise comparison and the conditional approach (analyzing mortality and duration of ventilation in a composite approach), the win ratio test performed better than all other tests in all but one scenario (Table 2). In the conflicting scenario, the hierarchical approach combined with the *t* test achieved the best performance. The truncated approach performed better in scenarios with weak mortality and strong duration and duration effects only. The best test results for each of the scenarios studied are described in table 3. The results of the reanalysis of two clinical trials are reported in table 4.

**Table 2:** Additional power calculations for different statistical tests with ventilator-free days on Day 28, mortality or duration of ventilation as the outcome and considering a composite approach

Effect		Mortality*(%)	Duration of ventilation in survivors*	Win ratio(%)	Conditional approach(%)		Truncated parametric§(%)	Truncated semiparametric§(%)
					Hierarchical t test†	Hierarchical Wilcoxon rank-sum test† ‡		
Mortality only								
	Treatment	15	7.0	58	5	5	75	75
	Control	25	7.0					
Strong mortality and weak duration								
	Treatment	15	6.0	90	39	31	89	89
	Control	25	7.0					
Moderate mortality and duration								
	Treatment	15	5.0	85	80	70	83	83
	Control	20	6.5					
Weak mortality and strong duration								
	Treatment	15	5.0	96	65	65	99	99
	Control	16	8.0					
Duration only								
	Treatment	15	5.0	95	52	52	99	99
	Control	15	8.0					
Conflicting								
	Treatment	15	6.5	12	79	67	74	75
	Control	20	5.0					

The test with the highest power in each scenario is highlighted in bold.

All results based on 3,000 simulated trials with 300 subjects in each of two treatment groups, a two-sided alternative hypothesis, and a type I error rate of α = 0.05.

* Mortality simulated according to a Bernoulli distribution and duration of ventilation in survivors according to an exponential distribution;

† if mortality numerically higher in treatment group, the trial is considered negative. If not, ventilator-free days on Day 28 for survivors was compared according to the specified test;

‡ normal approximation with continuity correction was used for the Wilcoxon rank-sum test;

§ the binary component and the continuous component are modeled separately, but just one single test for treatment effect was performed.

**Table 3:** Best test for each scenario assessed

Effect		Mortality(%)	Duration of ventilation in survivors	Best test	Power(%)
Mortality only					
	Treatment	15	7.0	Fisher's exact test*Gray's test†	84 78
	Control	25	7.0		
Strongmortalityandweakduration					
	Treatment	15	6.0	Gray's test Fine-Gray model	95 94
	Control	25	7.0		
Moderatemortalityandduration					
	Treatment	15	5.0	Wilcoxon rank-sum test/Cumulative logistic modelWin ratio	85 85
	Control	20	6.5		
Weakmortalityandstrongduration					
	Treatment	15	5.0	Truncated parametric or semiparametric Wilcoxon rank-sum test/Cumulative logistic model Win ratio	99 97 96
	Control	16	8.0		
Durationonly					
	Treatment	15	5.0	Truncated parametric or semiparametric Wilcoxon rank-sum test/Cumulative logistic model Win ratio	99 96 95
	Control	15	8.0		
Conflicting					
	Treatment	15	6.5	Hierarchical *t* test	79
	Control	20	5.0		

All results based on 3,000 simulated trials with 300 subjects in each of two treatment groups, a two-sided alternative hypothesis, and a type I error rate of α = 0.05.

* Outcome is mortality, duration of ventilation is ignored;

† when considering mortality and duration of ventilation.

**Table 4:** Alternative methods for ventilator-free days analysis using data from two different trials

			TEAM	SPICE III
Arms				
	Intervention		Early mobilization	Dexmedetomidine
	Control		Usual care	Usual care
Ventilator-free days on Day 28				
	Intervention		21 (8 - 25) 16.5 ± 9.9	23 (0 - 26) 16.5 ± 11.1
	Control		21 (11 - 25) 17.2 ± 9.3	22 (0 - 25) 16.2 ± 11.0
28-day mortality (%)				
	Intervention		16	24
	Control		11	24
Duration of ventilation in survivors, days				
	Intervention		5 (3 - 11) 8.4 ± 7.5	3 (1 - 7) 6.7 ± 13.2
	Control		6 (3 - 11) 8.6 ± 7.5	3 (2 - 8) 7.2 ± 11.6
Tests				
	Median regression (asymmetric Laplace)		MD, 0.60 (-0.77 - 1.99)	MD, 1.44 (0.60 - 2.27)
		p value	0.392	< 0.001
	Median regression (simplex)		MD, 0.00 (-1.47 - 1.47)	MD, 1.00 (0.39 - 1.61)
		p value	0.999	0.001
	Median regression (interior point)		MD, 0.00 (-1.47 - 1.47)	MD, 1.00 (0.39 - 1.60)
		p value	0.999	0.001
	Cumulative logistic regression model		COR, 0.94 (0.73 - 1.21)	COR, 1.09 (0.98 - 1.22)
		p value	0.661	0.112
	Win ratio		WR, 0.96 (0.80 - 1.14)	WR, 1.06 (0.98 - 1.14)
		p value	0.641	0.175
	Hierarchical *t*test		Not estimated	Not estimated
		p value	---	0.382
	Hierarchical Wilcoxon rank-sum test		Not estimated	Not estimated
		p value	---	0.010
	Truncated parametric		OR, 0.74 (0.51 - 1.08)	OR, 1.01 (0.88 - 1.17)
		p value	0.212	0.425
	Truncated semiparametric		OR, 0.74 (0.51 - 1.07)	OR, 1.01 (0.88 - 1.17)
		p value	0.213	0.426

MD - median difference; COR - common odds ratio; WR - win ratio; OR - odds ratio.95% confidence interval inside brackets. Data are reported as median (25th quartile - 75th quartile), mean ± standard deviation or percentage (%).

## DISCUSSION

In accordance with a previous paper,^(1)^ we found that the relative power of each statistical test was heavily dependent upon the magnitude of the treatment effect for the individual components of the composite score. While cumulative logistic regression, median regression and the win ratio displayed good power when the duration effect was dominant, none performed well when there was a mortality-only effect or when there were conflicting findings. These observations highlight the essential need to consider the individual components separately when analyzing composite scores.

## CONCLUSION

In this study, we describe the relative power of new methods for analyzing ventilator-free days in critical care research. Our data provide validation and guidance for the use of the cumulative logistic model, median regression, generalized pairwise comparisons, and the conditional and truncated approach in specific scenarios.
