# Supplementary material for: Alternative approaches to analyzing ventilator-free days, mortality and duration of ventilation in critical care research
Source: Crit Care Sci. 2024 May 7;36:e20240246en. doi: 10.62675/2965-2774.20240246-en (PMC11098075; doi:10.62675/2965-2774.20240246-en)
Supplement: Translation

# Translation: Abordagens alternativas para analisar dias livres de ventilador, mortalidade e duração da ventilação em estudos de cuidados intensivos

**DOI:** 10.62675/2965-2774.20240246-pt

**Authors:** Ary Serpa, Michael Bailey, Yahya Shehabi, Carol L Hodgson, Rinaldo Bellomo

**Affiliations:** 1 Melbourne Victoria Australia Australian and New Zealand Intensive Care Research Centre, School of Public Health and Preventive Medicine, Monash University - Melbourne, Victoria, Australia.; 2 São Paulo SP Brasil Departamento de Medicina Intensiva, Hospital Israelita Albert Einstein - São Paulo (SP), Brasil.; 3 Melbourne Victoria Australia Monash Health School of Clinical Sciences, Monash University - Melbourne, Victoria, Australia.; 4 Heidelberg VIC Australia Department of Intensive Care, Austin Hospital - Heidelberg VIC, Australia.

**Keywords:** Resultados de cuidados críticos, Métodos, Estatística, Respiração artificial, Cuidados críticos

## Abstract

**Objetivo:**

Discutir os pontos fortes e as limitações dos dias livres de ventilador e fornecer uma discussão abrangente dos diferentes métodos analíticos para analisar e interpretar esse desfecho.

**Métodos:**

Por meio de simulações, avaliou-se o poder de diferentes métodos analíticos, a saber: regressão quantílica (mediana), regressão logística cumulativa, comparação generalizada entre pares, abordagem condicional e abordagem truncada. No total, foram computadas 3.000 simulações de um estudo de dois braços com n = 300 por braço, usando uma hipótese alternativa bilateral e uma taxa de erro tipo I de α = 0,05.

**Resultados:**

Ao considerar o poder, a regressão mediana não teve bom desempenho em estudos em que o efeito do tratamento foi impulsionado principalmente pela mortalidade. A regressão mediana teve desempenho melhor em situações com efeito fraco na mortalidade, mas forte na duração, somente na duração e na mortalidade e duração moderadas. Verificou-se que a regressão logística cumulativa produziu um poder semelhante ao do teste de soma de postos de Wilcoxon em todos os cenários, sendo a melhor estratégia nos cenários de mortalidade e duração moderadas, mortalidade fraca e duração forte, e apenas duração.

**Conclusão:**

Neste estudo, descrevemos o poder relativo de novos métodos para analisar os dias livres de ventilador em estudos de cuidados intensivos. Nossos dados fornecem validação e orientação quanto ao uso do modelo logístico cumulativo, regressão mediana, comparações generalizadas entre pares e a abordagem condicional e truncada em cenários específicos.

## INTRODUÇÃO

O número de dias livres de ventilador (DLVs) é um dos vários desfechos de ausência de falência de órgãos comumente usados em estudos de cuidados intensivos, especialmente em estudos voltados a intervenções direcionadas ao sistema respiratório.^(1)^ Os DLVs representam desfecho composto que combina a mortalidade e a duração da ventilação em uma única variável, atenuando o efeito do risco concorrente de mortalidade. Uma das principais justificativas por trás dos DLVs é ter um desfecho contínuo que ofereça maior poder estatístico para detectar um efeito de tratamento do que apenas desfechos binários. Em um artigo recente, Yehya et al. forneceram uma estrutura completa para determinar quando e como usar os DLVs, com uma discussão abrangente sobre os diferentes métodos de análise e interpretação e o poder estatístico relativo de cada teste.^(1)^ Nesse sentido, estudos recentes também exploraram outros métodos de análise, a saber: regressão quantílica (mediana),^(2,3)^ regressão logística cumulativa,^(4,5)^ comparações generalizadas entre pares, incluindo o método *win ratio*,^(6)^ além de abordagens condicionais e truncadas.

Neste estudo, procuramos introduzir o conceito do efeito de distorção da percepção, discutir outros aspectos do uso de DLVs em estudos de cuidados intensivos e desenvolver trabalhos anteriores, considerando o poder relativo de outras abordagens. Além disso, foram testadas e descritas simulações de poder baseadas em um estudo anterior e métodos alternativos de análise.

## EFEITO DE DISTORÇÃO DA PERCEPÇÃO

O efeito de distorção da percepção está relacionado à maneira como os clínicos percebem e reagem de forma diferente aos achados de uma determinada intervenção, de acordo com a maneira como ela é apresentada. Por exemplo, consideramos uma intervenção que não afetou a mortalidade (idêntica em ambos os grupos), mas diminuiu a duração da ventilação em 1 dia em uma população de pacientes com uma duração média de ventilação de 2 dias. Assim, a duração média no grupo controle foi de 3 dias e, no grupo intervenção, foi de 2 dias. A maioria dos clínicos reagiria a esse achado como uma melhora substancial com implicações clínicas e práticas. Atualmente, esses pacientes são seguidos por 28 dias, e o desfecho dos DLVs é expresso como mediana, o que não seria influenciado de forma significativa nem mesmo por 20% de mortalidade. Os achados seriam 25 *versus* 26 DLVs.

Isso seria visto como algo trivial e, provavelmente, não provocaria a mesma reação. Na mente dos clínicos, o primeiro seria visto como uma evolução de 33%; o segundo seria visto como uma evolução de 3,8%. Essa distorção é ainda mais dramática se o seguimento for estendido para 90 ou 180 dias. Dessa forma, os DLVs podem distorcer a percepção e a reação a um efeito importante na duração da ventilação, resultando na rejeição e na negligência das terapias que obtiveram esses efeitos. Isso sugere que associar DLVs como desfecho com o desfecho secundário de duração da ventilação em sobreviventes pode ser vantajoso na ausência de um aumento numérico na mortalidade em pacientes que recebem a intervenção avaliada.

Na medicina, os vieses cognitivos, como a distorção da percepção, resultam em erros de diagnóstico e atrasos na aceitação de novas descobertas científicas. Por exemplo, apesar das boas evidências que sugerem o impacto dos anticorpos séricos de antígeno leucocitário humano (HLA - *human leukocyte antigen*) nos desfechos de transplantes, as recomendações consensuais para a inclusão rotineira de testes de anticorpos HLA como parte do monitoramento pós-transplante não são feitas há mais de 30 anos.^(7)^ Além disso, as respostas à detecção de anticorpos HLA no soro ainda variam, e, há mais de 40 anos, não se chega a uma recomendação consensual para o tratamento de rotina. Esse atraso na aceitação do papel dos anticorpos HLA na rejeição de transplantes é um exemplo de um viés cognitivo, como o viés de confirmação ou a distorção da percepção dos achados da pesquisa.^(7)^

## ABORDAGENS ALTERNATIVAS PARA ANÁLISE

### Regressão quantílica (mediana)

Desde sua criação em 1978,^(8)^ a regressão quantílica ou mediana tornou-se uma ferramenta importante na pesquisa médica para a análise de dados não paramétricos e ofereceu a vantagem semelhante de permitir o ajuste de covariáveis e estimativas de efeito de tratamento com intervalos de confiança. No entanto, devido à natureza composta e de classificação dos DLVs, podem ocorrer diferenças fundamentais nos desfechos mesmo que os valores medianos sejam idênticos.^(1)^ Além disso, o componente de mortalidade é extremamente importante, mas tem pouco efeito sobre a mediana. Assim, o poder da regressão mediana é provavelmente muito influenciado pelo componente que impulsiona o efeito dos DLVs: a duração da ventilação ou a mortalidade.

A regressão quantílica tem muitas vantagens, mas sua principal desvantagem é que seus parâmetros são mais difíceis de estimar do que os dos métodos mais tradicionais (por exemplo, regressão gaussiana ou generalizada). As inferências dessa regressão quantílica podem ser complicadas porque os estimadores dos coeficientes não estão disponíveis em uma forma fechada.^(9)^ A maneira mais comum de resolver esse problema é usar um algoritmo de otimização linear com intervalos de confiança baseados em aproximações lineares por partes.^(8)^ Outra maneira possível é usar algoritmos de reforço. No entanto, a implementação de valores de p e intervalos de confiança dos parâmetros de regressão estimados não é simples.^(10)^ Por fim, um algoritmo desenvolvido mais recentemente foi baseado na verossimilhança Laplace assimétrica.^(11)^Assim, a estimativa pode ser altamente dependente do método escolhido. Esse método de análise foi usado recentemente em dois estudos clínicos randomizados na área de cuidados intensivos.^(2,3)^

### Regressão logística cumulativa

A regressão logística cumulativa considera a classificação e a estrutura ordinal dos DLVs.^(4,5)^ Nesse modelo, as probabilidades logarítmicas cumulativas são modeladas de forma que um parâmetro maior que zero reflita um aumento nas probabilidades cumulativas do desfecho DLVs, o que implica benefício. Possivelmente, a vantagem desse modelo é que, com a amostragem multinomial de indivíduos independentes, a estatística do teste de pontuação do modelo é semelhante à estatística do teste de soma de postos de Wilcoxon,^(12)^ um dos testes mais poderosos para analisar os DLVs em diversos cenários.^(1)^ No entanto, com o modelo logístico cumulativo, é possível ajustar ainda mais os fatores de confusão e extrair uma estimativa de efeito com um intervalo de confiança. A possível desvantagem é que o modelo pressupõe efeitos proporcionais em toda a escala ordinal de DLVs. Isso é chamado de "pressuposto de chances proporcionais" ou "pressuposto de regressão paralela". Esse método de análise foi aplicado recentemente em dois ensaios clínicos randomizados na área de cuidados intensivos.^(4,5)^

### Comparação generalizada entre pares

O número de DLVs é um desfecho composto que considera o número de mortes e a duração da ventilação no cálculo. Na prática clínica, a importância da morte é muito maior do que a da duração da ventilação. Ao comparar dois pacientes submetidos a um novo tratamento ou estratégia, é razoável priorizar o efeito sobre a morte antes do efeito sobre a duração da ventilação. Portanto, com base nesse raciocínio, primeiro é preciso determinar se um paciente morreu antes de avaliar a duração da ventilação. Se isso não for conhecido, deve-se determinar qual paciente teve uma duração de ventilação mais longa. Se ambos os pacientes sobreviveram e tiveram a mesma duração de ventilação, eles seriam considerados empatados. Esse tipo de análise é possível de várias maneiras, incluindo a comparação de pares combinados (usando uma abordagem de *win ratio*)^(13)^ ou pares não combinados (usando o método descrito por Finkelstein e Schoenfeld).^(14)^ Esse método de análise foi usado recentemente em um ensaio clínico randomizado em cuidados intensivos.^(6)^

### Abordagem condicional

Com base na lógica descrita, que prioriza a morte em relação à duração da ventilação, outra possível estratégia é usar uma abordagem condicional. Essa abordagem segue uma sequência de testes fixos predefinidos com base em informações clínicas.^(15)^ Com essa estratégia, se a intervenção estudada simplesmente resultar em uma porcentagem numericamente maior de mortes do que nos controles, nenhuma outra avaliação é feita, e o estudo é julgado como *neutro* ou *negativo*, dependendo da magnitude do efeito sobre a mortalidade. Entretanto, se a intervenção resultar em uma taxa de mortalidade menor, a duração da ventilação nos sobreviventes será comparada entre os grupos estudados por meio de testes tradicionais. Esse procedimento fundamenta-se na ideia de que uma intervenção que leva a um aumento numérico na mortalidade, mesmo que não seja estatisticamente significativa, é de menor importância e provavelmente não seria implementada na assistência clínica, mesmo que resultasse em uma duração mais curta da ventilação. No presente estudo, usamos um teste *t* hierárquico e um teste hierárquico de soma de postos de Wilcoxon como abordagens condicionais.

### Abordagem truncada

Recentemente, foi relatado um novo teste de alto poder para desfechos contínuos truncados por morte.^(16)^ Essa abordagem incorpora o conceito de que esse tipo de desfecho é, de fato, bidimensional e que o desfecho combinado construído segue uma distribuição de mistura contínua-singular. Considerando essa premissa, os autores sugerem que essa distribuição incomum é a razão pela qual não se pode recorrer a testes não paramétricos de soma de postos de Wilcoxon. Isso ocorre porque o componente singular da distribuição do desfecho combinado será reduzido a simples empates. A esse respeito, o tratamento de empates no *software* estatístico padrão varia e é opaco. Entretanto, o tratamento de empates não é o principal motivo pelo qual o Wilcoxon sofre perda de poder. O principal motivo é que a hipótese nula nesses testes do tipo Wilcoxon (dominação estocástica) não lida com o fato empírico de que os tratamentos podem influenciar a mortalidade e a duração da ventilação de forma diferente.

Os autores propõem modelar o componente binário (ou seja, a sobrevida) e a parte contínua (ou seja, os dias efetivamente livres de ventilador) separadamente, porém realizar um único teste para nenhum efeito do tratamento em nenhum deles. Essa abordagem fornece um único valor de p para a hipótese de nenhum efeito do tratamento nos dias prolongados livres de ventilador em que a morte recebe a pontuação mais baixa possível. Para acomodar a possível não normalidade dos DLVs registrados, descrevemos os testes paramétricos e semiparamétricos.

## MÉTODOS

Para manter a consistência e facilitar a comparação, adotamos a mesma estratégia implementada anteriormente.^(1)^ No total, foram computadas 3.000 simulações de um estudo de dois braços com n = 300 por braço, usando uma hipótese alternativa bilateral e uma taxa de erro tipo I de α = 0,05. A mortalidade foi simulada segundo uma distribuição de Bernoulli, e a duração da ventilação entre os sobreviventes foi simulada conforme uma distribuição exponencial. Todas as mortes foram atribuídas a zero DLVs. Aos pacientes com tempo de ventilação superior a 28 dias, foram atribuídos zero DLVs, enquanto para os demais pacientes, o tempo de ventilação foi calculado como 28. Conforme descrito,^(1)^ consideramos uma série de cenários com efeitos de tratamento variáveis para mortalidade e duração da ventilação. Para fins de comparação e validade, replicamos os cálculos de poder realizados anteriormente,^(1)^ incluindo o modelo de risco concorrente de Fine-Gray, o teste de Gray, o teste de soma de postos de Wilcoxon, o teste *t* de Student e o teste exato de Fisher. Testamos três algoritmos diferentes na regressão mediana: distribuição Laplace assimétrica, simplex e ponto interior. Na regressão logística cumulativa, os DLVs foram arredondados para uma casa decimal, para melhorar a eficiência computacional. O método de *win ratio* foi calculado com a morte priorizada ante os DLVs em sobreviventes e com a distribuição de amostras grandes de determinadas estatísticas U multivariadas de múltiplas amostras.

Todas as simulações foram realizadas no R v.4.0.2, e os seguintes pacotes foram usados além do programa principal: lqmm,^(11)^ quantreg,^(17)^ cmprsk,^(18)^ ordinal^(19)^ e WinRatio.^(20)^ Para ilustrar os métodos estudados para analisar DLVs, foram realizados dois ensaios clínicos: SPICE III^(3)^ e TEAM.^(21)^

## RESULTADOS

Ao considerar o poder, a regressão mediana não teve um bom desempenho em estudos em que o efeito do tratamento foi impulsionado principalmente pela mortalidade (Tabela 1). A regressão mediana teve desempenho melhor em situações com efeito fraco na mortalidade, mas forte na duração, somente na duração e na mortalidade e duração moderadas. Entretanto, a regressão mediana não teve desempenho melhor do que o teste de soma de postos de Wilcoxon em nenhum desses cenários. O algoritmo subjacente também desempenha papel importante na determinação do poder da regressão mediana, e o algoritmo de "ponto interior" tem o maior poder, enquanto o algoritmo Laplace assimétrico foi o menos potente. O único cenário em que a regressão mediana apresentou o maior poder com o algoritmo Laplace assimétrico foi o cenário conflitante.

**Tabela 1:** Cálculos de poder de diferentes testes estatísticos com dias livres de ventilador aos 28 dias como desfecho

Efeito		Mortalidade*(%)	Duração da ventilação em sobreviventes*	Modelo Fine-Gray(%)	Teste de Gray(%)	Teste de soma de postos de Wilcoxon†(%)	Teste t de Student(%)	Teste exato de Fisher‡(%)	Regressão mediana§a(%)			Modelo logístico cumulativo
									Laplace assimétrico	Simplex	Ponto interior	
Apenas mortalidade												
	Tratamento	15	7,0	77	78	56	72	84	4	12	20	56
	Controle	25	7,0									
Mortalidade forte e duração fraca												
	Tratamento	15	6,0	94	95	89	94	84	36	52	52	89
	Controle	25	7,0									
Mortalidade e duração moderadas												
	Tratamento	15	5,0	78	80	85	81	32	24	72	80	85
	Controle	20	6,5									
Mortalidade fraca e duração forte												
	Tratamento	15	5,0	83	84	97	90	5	44	88	92	97
	Controle	16	8,0									
Apenas duração												
	Tratamento	15	5,0	77	78	96	86	4	36	88	92	96
	Controle	15	8,0									
Conflitante												
	Tratamento	15	6,5	5	5	14	5	32	64	28	24	14
	Controle	20	5,0									

O teste com maior poder em cada cenário está destacado em negrito.

Todos os resultados baseiam-se em 3.000 testes simulados com 300 indivíduos em cada um dos dois grupos intervenção, uma hipótese alternativa bilateral e uma taxa de erro tipo I de α = 0,05.

* Mortalidade simulada de acordo com uma distribuição de Bernoulli e duração da ventilação em sobreviventes conforme uma distribuição exponencial;

† a aproximação normal com correção de continuidade foi usada para o teste de soma de postos de Wilcoxon;

‡ o desfecho é a mortalidade, a duração da ventilação é ignorada;

§ todos os valores de p extraídos por meio de *bootstrap* com 1.000 amostras.

Ao considerar o poder, verificou-se que a regressão logística cumulativa produziu poder semelhante ao teste de soma de postos de Wilcoxon em todos os cenários, sendo a melhor estratégia para os cenários de mortalidade e duração moderadas, mortalidade fraca e duração forte, e somente duração.

Ao considerar a comparação generalizada entre pares e a abordagem condicional (analisando a mortalidade e a duração da ventilação em uma abordagem composta), o teste de *win ratio* teve um desempenho melhor do que todos os outros testes em todos os cenários, exceto um (Tabela 2). No cenário conflitante, a abordagem hierárquica combinada com o teste *t* obteve o melhor desempenho. A abordagem truncada teve melhor desempenho em cenários com mortalidade fraca e forte duração e somente efeitos de duração. Os melhores resultados de teste para cada um dos cenários estudados estão descritos na tabela 3. Os resultados da reanálise de dois estudos clínicos são relatados na tabela 4.

**Tabela 2:** Outros cálculos de poder de diferentes testes estatísticos com dias livres de ventilador aos 28 dias, mortalidade ou duração da ventilação como desfecho e considerando-se uma abordagem composta

Efeito		Mortalidade*(%)	Duração da ventilação em sobreviventes*	Win ratio(%)	Abordagem condicional(%)		Paramétrico truncado§ (%)	Semiparamétrico truncado§(%)
					Teste t hierárquico†	Teste hierárquico de soma de postos de Wilcoxon† ‡		
Apenas mortalidade								
	Tratamento	15	7,0	58	5	5	75	75
	Controle	25	7,0					
Mortalidade forte e duração fraca								
	Tratamento	15	6,0	90	39	31	89	89
	Controle	25	7,0					
Mortalidade e duração moderadas								
	Tratamento	15	5,0	85	80	70	83	83
	Controle	20	6,5					
Mortalidade fraca e duração forte								
	Tratamento	15	5,0	96	65	65	99	99
	Controle	16	8,0					
Apenas duração								
	Tratamento	15	5,0	95	52	52	99	99
	Controle	15	8,0					
Conflitante								
	Tratamento	15	6,5	12	79	67	74	75
	Controle	20	5,0					

O teste com a maior poder em cada cenário está destacado em negrito.

Todos os resultados baseiam-se em 3.000 testes simulados com 300 indivíduos em cada um dos dois grupos intervenção, uma hipótese alternativa bilateral e uma taxa de erro tipo I de α = 0,05.

* Mortalidade simulada de acordo com uma distribuição de Bernoulli e duração da ventilação nos sobreviventes de acordo com uma distribuição exponencial;

† se a mortalidade for numericamente maior no grupo intervenção, o ensaio é considerado negativo. Caso contrário, os dias livres de ventilador no 28º dia nos sobreviventes foram comparados conforme o teste específico;

‡ a aproximação normal com correção de continuidade foi usada para o teste de soma de postos de Wilcoxon;

§ o componente binário e o componente contínuo são modelados separadamente, mas foi realizado apenas um único teste para o efeito do tratamento.

**Tabela 3:** Melhor teste para cada cenário avaliado

Efeito		Mortalidade(%)	Duração da ventilação em sobreviventes	Melhor teste	Poder(%)
Apenas mortalidade					
	Tratamento	15	7,0	Teste exato de Fisher* Teste de Gray†	84 78
	Controle	25	7,0		
Mortalidade forte e duração fraca					
	Tratamento	15	6,0	Teste de Gray Modelo Fine-Gray	95 94
	Controle	25	7,0		
Mortalidade e duração moderadas					
	Tratamento	15	5,0	Teste de soma de postos de Wilcoxon/Modelo logístico cumulativo Win ratio	85 85
	Controle	20	6,5		
Mortalidade fraca e duração forte					
	Tratamento	15	5,0	Paramétrico ou semiparamétrico truncado Teste de soma de postos de Wilcoxon/Modelo logístico cumulativo *Win ratio*	99 97 96
	Controle	16	8,0		
Apenas duração					
	Tratamento	15	5,0	Paramétrico ou semiparamétrico truncado Teste de soma de postos de Wilcoxon/Modelo logístico cumulativo *Win ratio*	99 96 95
	Controle	15	8,0		
Conflitante					
	Tratamento	15	6,5	Teste *t* hierárquico	79
	Controle	20	5,0		

Todos os resultados baseiam-se em 3.000 testes simulados com 300 indivíduos em cada um dos dois grupos intervenção, uma hipótese alternativa bilateral e uma taxa de erro tipo I de α = 0,05.

* O desfecho é a mortalidade, a duração da ventilação é ignorada;

† ao considerar a mortalidade e a duração da ventilação.

**Tabela 4:** Métodos alternativos para análise de dias livres de ventilador usando dados de dois estudos diferentes

			TEAM	SPICE III
Braços				
	Intervenção		Mobilização precoce	Dexmedetomidina
	Controle		Cuidados habituais	Cuidados habituais
Dias livres de ventilador aos 28 dias				
	Intervenção		21 (8 - 25) 16,5 ± 9,9	23 (0 - 26) 16,5 ± 11,1
	Controle		21 (11 - 25) 17,2 ± 9,3	22 (0 - 25) 16,2 ± 11,0
Mortalidade aos 28 dias (%)				
	Intervenção		16	24
	Controle		11	24
Duração da ventilação nos sobreviventes, dias				
	Intervenção		5 (3 - 11) 8,4 ± 7,5	3 (1 - 7) 6,7 ± 13,2
	Controle		6 (3 - 11) 8,6 ± 7,5	3 (2 - 8) 7,2 ± 11,6
Testes				
	Regressão mediana (Laplace assimétrico)		DM, 0,60 (-0,77 - 1,99)	DM, 1,44 (0,60 - 2,27)
		Valor de p	0,392	< 0,001
	Regressão mediana (simplex)		DM, 0,00 (-1,47 - 1,47)	DM, 1,00 (0,39 - 1,61)
		Valor de p	0,999	0,001
	Regressão mediana (ponto interior)		DM, 0,00 (-1,47 - 1,47)	DM, 1,00 (0,39 - 1,60)
		Valor de p	0,999	0,001
	Modelo de regressão logística cumulativa		RCC, 0,94 (0,73 - 1,21)	RCC, 1,09 (0,98 - 1,22)
		Valor de p	0,661	0,112
	*Win ratio*		WR, 0,96 (0,80 a 1,14)	WR, 1,06 (0,98 - 1,14)
		Valor de p	0,641	0,175
	Teste *t* hierárquico		Não estimado	Não estimado
		Valor de p	---	0,382
	Teste hierárquico de soma de postos de Wilcoxon		Não estimado	Não estimado
		Valor de p	---	0,010
	Paramétrico truncado		RC, 0,74 (0,51 - 1,08)	RC, 1,01 (0,88 - 1,17)
		Valor de p	0,212	0,425
	Semiparamétrico truncado		RC, 0,74 (0,51 - 1,07)	RC, 1,01 (0,88 - 1,17)
		Valor de p	0,213	0,426

DM - diferença mediana; RCC - razão de chances comum; WR - win ratio; RC - razão de chances. Intervalo de confiança de 95% entre parênteses. Os dados apresentam-se como mediana (25º quartil - 75º quartil), média ± desvio padrão ou porcentagem (%).

## DISCUSSÃO

De acordo com estudo anterior,^(1)^ descobrimos que o poder relativo de cada teste estatístico dependia muito da magnitude do efeito do tratamento nos componentes individuais do escore composto. Embora a regressão logística cumulativa, a regressão mediana e a *win ratio* tenham apresentado bom poder quando o efeito da duração era dominante, nenhum deles teve um bom desempenho quando houve um efeito apenas de mortalidade ou achados conflitantes. Essas observações destacam a necessidade essencial de considerar os componentes individuais separadamente ao analisar os escores compostos.

## CONCLUSÃO

Neste trabalho, descrevemos o poder relativo de novos métodos para analisar dias sem ventilador em estudos de cuidados intensivos. Nossos dados fornecem validação e orientação para o uso do modelo logístico cumulativo, regressão mediana, comparações pareadas generalizadas e abordagem condicional e truncada em cenários específicos.
